# Effects of Microscopic Properties on Macroscopic Thermal Conductivity for Convective Heat Transfer in Porous Materials

**DOI:** 10.3390/mi12111369

**Published:** 2021-11-07

**Authors:** Mayssaa Jbeili, Junfeng Zhang

**Affiliations:** Bharti School of Engineering and Computer Science, Laurentian University, 935 Ramsey Lake Road, Sudbury, ON P3E 2C6, Canada; mjbeili@laurentian.ca

**Keywords:** heat transfer, porous media, pore-scale modeling, boundary condition, thermal conductivity, porosity, conjugate interface, aspect ratio

## Abstract

Porous materials are widely used in many heat transfer applications. Modeling porous materials at the microscopic level can accurately incorporate the detailed structure and substance parameters and thus provides valuable information for the complex heat transfer processes in such media. In this study, we use the generalized periodic boundary condition for pore-scale simulations of thermal flows in porous materials. A two-dimensional porous model consisting of circular solid domains is considered, and comprehensive simulations are performed to study the influences on macroscopic thermal conductivity from several microscopic system parameters, including the porosity, Reynolds number, and periodic unit aspect ratio and the thermal conductance at the solid–fluid interface. Our results show that, even at the same porosity and Reynolds number, the aspect ratio of the periodic unit and the interfacial thermal conductance can significantly affect the macroscopic thermal behaviors of porous materials. Qualitative analysis is also provided to relate the apparent thermal conductivity to the complex flow and temperature distributions in the microscopic porous structure. The method, findings and discussions presented in this paper could be useful for fundamental studies, material development, and engineering applications of porous thermal flow systems.

## 1. Introduction

Fluid flows and the associated heat transfer in porous materials have attracted great interest over the decades for their scientific and practical importance. With the large contact area between the solid matrix and the coolant fluid, heat transfer performance is significantly enhanced in such porous materials. Relevant applications can be found in various industrial areas, including catalytic beds for chemical reactions, water treatment, nanofluids, heat exchangers, heat sinks and thermal energy storage systems [1,2,3,4,5,6,7,8]. Extensive experimental and theoretical investigations have been conducted to study the heat transfer performance in porous materials [9,10,11]. In addition to the high cost and long time duration, experimental studies are not able to reveal the flow and temperature distributions in the complex microscopic porous geometry, while such information is crucial for understanding the mechanism and relationships among various factors involved. On the other hand, theoretical analysis is limited to simple geometry structures and idealized flow situations, and such conditions can hardly be satisfied in realistic systems. Fortunately, with the advances in computer and software technologies, numerical simulations have been proved to be useful for various complex systems, including flows and heat transfer in porous media [12,13,14,15,16]. In the literature, there exist two typical approaches for modeling the flow and heat transfer in porous media: the continuum and the pore-scale approaches. The first method treats the porous materials as continuum media and solves the macroscopic flow and energy equations with apparent parameters [17,18]. Despite the model’s simplicity and computational efficiency, this approach relies on the accuracy of those apparent parameters and also cannot incorporate the specific microscopic structure and material thermophysical properties in the porous media. On the other hand, the pore-scale method solves the flow and energy equations with the microscopic structure and properties of the matrix material considered explicitly [19,20]. Pore-scale simulations can provide detailed flow and thermal fields at the microscopic level, and apparent properties can be obtained from these microscopic distributions for macroscopic analysis [21,22,23]. In recent years, the lattice Boltzmann method (LBM) has become especially popular in such simulations, mainly for its convenience in dealing with complex boundary geometry [22,23,24,25].

With the microscopic porous structure modeled explicitly, the physical scale of pore-scale simulations is limited to small sizes due to the large computation demand. For this reason, pore-scale simulations typically work with a small unit and assume that such identical units are repeated in space. To solve the flow and energy equations in a periodic unit, appropriate boundary conditions are necessary on the side surfaces of this computational unit. Such side surfaces are simply for computational convenience and they are not physical surfaces or boundaries; therefore, definite conditions on such surfaces are not available. To create a thermal gradient, previous studies are usually assigned constant temperatures on two opposite unit surfaces and assumed adiabatic (no heat flux) conditions on other side walls [24,25,26,27]. This treatment has been examined recently and significant concerns have been raised in terms of its validity and accuracy [28]. Few studies treated the unit surface conditions more reasonably by considering the periodic relations of temperature, however, the temperature or heat flux on the fluid-solid interface was fixed at constant values [29,30,31]. Clearly this is physically not true: the thermal condition (temperature and heat flux) at the microscopic fluid-solid interface is determined by the particular local flow and thermal situations and it cannot be specified in advance. The interfacial thermal resistance may also exist at the fluid-solid interface in porous materials [32] or the constituent interface in composite materials [33], and this feature has typically been neglected in previous studies. Furthermore, previous studies usually only considered situations with the flow along the temperature gradient direction [22,25,30], whereas in many experimental setups and industrial applications the temperature gradient is mainly in the orthogonal plane to the fluid flow [34,35,36].

In this paper, we study the convective heat transfer in porous materials using the recent generalized periodic boundary method for thermal flows by Jbeili and Zhang [28]. This boundary method was established with rigorous mathematical justifications and validated by carefully designed numerical tests. Using this method, extensive simulations are conducted based on a two-dimensional (2D) porous model of circular solid particles. Unlike previous studies usually focusing on the porosity and Reynolds number effects, we also investigate the influences of the unit aspect ratio and the interface conductance on the macroscopic thermal performances of porous flows. Our results show that, even with the same porosity and Reynolds number, the unit aspect ratio can greatly affect the apparent thermal conductivity at the microscopic level. It is also interesting to observe that a weaker interfacial conductance can reduce the tortuosity conductivity but increase the dispersion conductivity, and that the dispersion conductivity can respond to the porosity change in a non-monotonic fashion. In addition, qualitative discussions are provided to relate the macroscopic conductivity coefficients to the microscopic flow and thermal fields for a better understanding of the complex nature of porous thermal flows.

## 2. Governing Equations and Boundary Conditions for Periodic Unit

**Figure 1:**
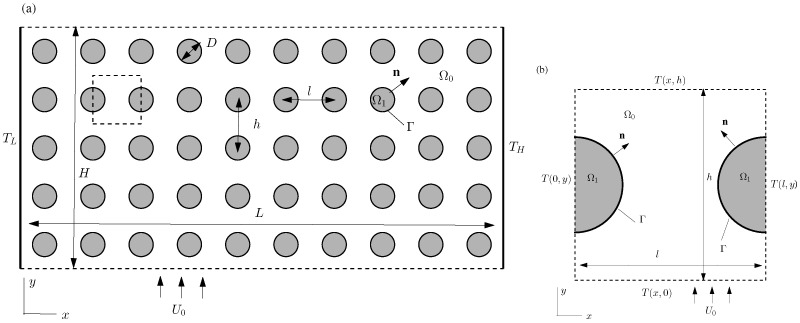
Schematic representations of the 2D porous model used in this study (**a**) and one periodic unit considered in pore-scale simulations (the dashed box in (**a**), and with more details in (**b**). The key parameters involved in the model description are also labeled, and more details can be found in the text.

For simplicity and clarity, in this study we consider the 2D porous model with circular solid domains as shown in Figure 1a. The fluid flows vertically from the bottom to the top, while the thermal gradient is imposed in the horizontal direction. The governing equations for this thermal flow system include the continuity equation, Equation (1), and the Navier–Stokes equation Equation (2) for the fluid domain $\Omega_{0}$:

(1) ∇·u=0,

(2) $$\rho \left(\frac{\partial u}{\partial t}+u\cdot \nabla u\right)=-\nabla p+\mu \nabla^{2}u+f;$$

and the energy equation

$$\frac{\partial T_{f}}{\partial t}+u\cdot \nabla T_{f}=\alpha_{f}\nabla^{2}T_{f},\;in\;\Omega_{0},$$

(3) $$\frac{\partial T_{s}}{\partial t}=\alpha_{s}\nabla^{2}T_{s},\;in\;\Omega_{1},$$

for the fluid ($\Omega_{0}$) and solid ($\Omega_{1}$) domains, respectively [37]. In these equations, ρ represents the fluid density, u is the fluid velocity, *p* is the pressure, μ is the dynamic viscosity of fluid, f is the body force on the fluid, *T* is the temperature, and α is the thermal diffusivity. The subscripts ${}_{f}$ and ${}_{s}$ for *T* and α denote the fluid and solid domains respectively. On the fluid-solid interface Γ, the no-slip boundary condition is applied for the fluid flow

(4) u=0,onΓ,

to incorporate the possible thermal resistance and therefore the temperature discontinuity at the solid–fluid interface Γ, a general conjugate condition for temperature is given as [38,39,40]:

(5) $$q=C\left(T_{f}-T_{s}\right)=K_{s}\frac{\partial T_{s}}{\partial n}=K_{f}\frac{\partial T_{f}}{\partial n},\;on\;\Gamma .$$

Here, *q* is the heat flux along the local normal direction n, which points from Domain $\Omega_{1}$ toward Domain $\Omega_{0}$ (Figure 1). *C* is the interface conductance, and $K_{f}$ and $K_{s}$ are the thermal conductivities of the fluid and solid substances, respectively.

In the pore-scale approach, only one periodic unit of the porous material is considered in the simulation (Figure 1). To solve the governing equations in this periodic unit, appropriate boundary conditions are required for the side boundaries of the periodic unit. These side boundaries are virtual but not real surfaces, and thus physical constraints on such boundaries are not directly available. For flows in such periodic structures with negligible fluid property changes, it has been well recognized that the flow field would be identical in each periodic unit [21,41,42,43]:

(6) u(x+ml,y+nh)=u(x,y),

where *l* and *h* are the unit length and height (Figure 1), and *m* and *n* are arbitrary integers. Based on previous practices in periodic thermal flow simulations [28,41,42,43,44,45], the following relationship is proposed for the temperature field among periodic units in Figure 1a:

(7) $$T\left(x+ml,y+nh\right)=T\left(x,y\right)+mlT_{g}^{\prime }.$$

Here, $T_{g}^{\prime }$ is the global thermal gradient in the horizontal direction, and it can be obtained from the temperature difference $T_{H}-T_{L}$ and the distance *L* between the locations where these two temperatures $T_{L}$ and $T_{H}$ are imposed (see Figure 1a) as $T_{g}^{\prime }=\left(T_{H}-T_{L}\right)/L$.

Assume u(x,y) and T(x,y) are the correct flow and temperature solutions in one periodic unit (0≤x≤l and 0≤y≤h), it can be readily shown that the velocity from Equation (6) and temperature from Equation (7) satisfy all the governing equations and boundary conditions given in Equations (1)–(5), and thus they are the correct solutions for the unit of ml≤x≤(m+1)l and nh≤y≤(n+1)h. Therefore, in pore-scale simulations, one can simulate the flow and thermal fields in one periodic unit as shown in Figure 1b, and the solutions can be extended to other units according to Equations (6) and (7). These relations can then be used to establish correct boundary conditions for the side surfaces of the periodic unit. For example, the right boundary x=l for the unit shown in Figure 1b actually is also the left boundary of the next unit on its right. According to Equations (6) and (7) with m=1 and n=0, we have the left-right boundary relations for the periodic unit as:

(8) $$u\left(l,y\right)=u\left(0,y\right),\;T\left(l,y\right)=T\left(0,y\right)+lT_{g}^{\prime }.$$

Similarly, the top-bottom boundary relations for flow and temperature are expressed as:

(9) u(x,h)=u(x,0),T(x,h)=T(x,0).

These relations are similar to those used by Kuwahara et al. [21]; however, no mathematical justifications and numerical validations were provided there. In addition to these boundary relations, due to the linearity and homogeneity of the governing equations and boundary conditions, extra anchoring conditions are necessary to ensure the numerical convergence [39,41,42]. For our current system in Figure 1a, the following conditions are adopted in our next simulations:

(10) $$\frac{1}{l}\int_{0}^{l}u\left(x,0\right)dx=U_{0},\;\frac{1}{h}\int_{0}^{h}T\left(0,y\right)dy=T_{0};$$

where $U_{0}$ is the mean velocity through the porous medium and $T_{0}$ is the mean temperature at the left unit boundary. The mean velocity $U_{0}$ is related to the Reynolds number Re of the system (to be defined later), while the mean temperature $T_{0}$ can be set at an arbitrary value and it has no impact on the result analysis and interpretation.

## 3. Numerical Validation of the Periodic Relations among Units

To further verify the flow and temperature relations among units given in Equations (6) and (7), we conduct a direct numerical simulation for the system in Figure 1a. This simulation is also helpful to illustrate our concerns with the periodic unit boundary treatments used in previous studies. The diameter of the solid domains is D=50 and they are arranged as a square array with l=h=2D. The porosity of this model medium is thus ϵ=π/16. The rectangular simulation domain has a length of L=20D in the horizontal direction and a hight of H=10D in the vertical direction, and thus the simulation domain consists 10×5=50 identical periodic units as shown in Figure 1b. Constant temperatures are imposed on the left and right boundaries: $T_{L}=0$ at x=0 and $T_{H}=1$ at x=L, resulting a global thermal gradient of $T_{g}^{\prime }=\left(T_{H}-T_{L}\right)/L$ = $10^{-3}$. The classical periodic condition is imposed on the top and bottom boundaries for both flow velocity and temperature, and on the left and right boundaries for the fluid flow: u(0,y)=u(L,y), u(x,0)=u(x,H), and T(x,0)=T(x,H). Please note that the temperature relation Equation (7) is not involved in this validation simulation. A body force f in the upward direction is utilized to generate the flow, and its magnitude is adjusted to obtain the Reynolds number $Re=\rho U_{0}\sqrt{lh}/\mu =50$. Here we use the geometric mean value of the periodic unit length *l* and height *h* for the Reynolds number Re, and this choice is made for our convenience in examining the effect of the aspect ratio β=h/l in Section 4.1. The Prandtl number of the fluid is 0.7. The thermal conductivities are set as $K_{f}=0.2$ for fluid and $K_{s}=10K_{f}=2$ for solid. A relatively large interface conductance value C=10 is adopted to minimize the temperature discontinuity across the interface. The lattice Boltzmann method (LBM) with the D2Q9 (2D and with nine lattice velocities) lattice structure is used to solve the flow and thermal fields [44,46,47,48] for all calculations in this paper, and the counter-extrapolation method [49] is adopted for the conjugate thermal condition on domain interfaces. As in general LBM studies [16,22,31,48,49,50], all quantities provided above and in the later result presentation are non-dimensional based on LBM simulation units (for example, length in the lattice grid resolution, and time in the simulation time step).

**Figure 2:**
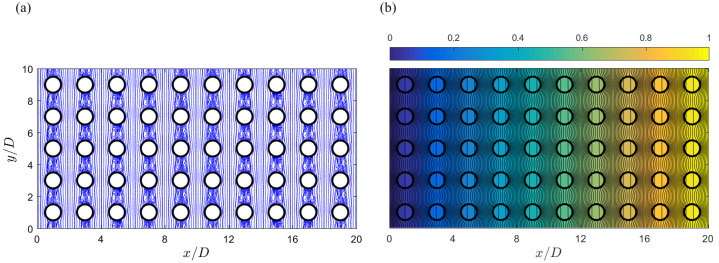
Flow (**a**) and temperature (**b**) distributions obtained from the direct simulation for the 2D porous model system in Figure 1a.

The calculated flow and temperature fields from this direct simulation are displayed in Figure 2. The flow pattern in Figure 2a appears identical in each periodic unit. For the temperature in Figure 2b, it can be seen that the temperature increases in general along the horizontal direction, agreeing with the global thermal gradient generated from the two boundary temperatures $T_{L}=0$ at the left and $T_{H}=1$ at the right. In Figure 2b, neither the temperature nor the heat flux are constant along the solid–fluid interface (edges of the circular patches), meaning that the constant temperature or flux assumptions for the interfaces in previous studies [22,30,31] are not valid. The wider gaps between isotherm contours in the solid domain imply smaller temperature gradients there, and this is consistent with the larger solid conductivity used in this simulation. With a relatively larger conductance value for the solid–fluid interface, no apparent discontinuity in temperature can be observed across the interfaces.

**Figure 3:**
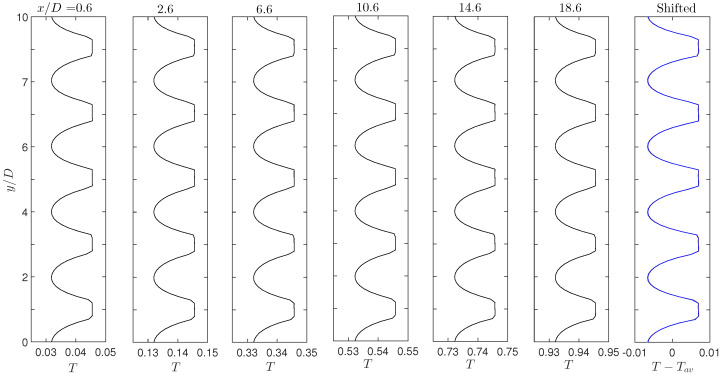
Temperature profiles along the vertical direction at different horizontal positions (labeled on top) from the direct simulation results in Figure 2b. The last panel on the right collects all the temperature profiles in other panels, however shifted by their individual mean temperatures. These six shifted profiles become identical and completely overlap each other, confirming the thermal relation Equation (7) among periodic units along the horizontal direction.

The apparently linear increase in temperature and the similarity in local isotherm contours in Figure 2b appear rational according to the temperature relation Equation (7). Moreover, T∼y profiles at six horizontal locations are plotted in Figure 3, one in each panel from the left and with the *x* positions labeled on top. These horizontal positions are selected at the same relative locations to the nearby solid columns (0.4D to the left of the patch centers). The flat segments on these curves occur in the solid domains, and they are due to the vertical isotherm lines there. The strong variations in these profiles clearly show that in general one should not assign constant temperatures on the periodic unit boundaries, as done in [24,25,26]. According to Equation (7), these temperature profiles should have the same variation features along the vertical direction, except different offset values. This is apparently true by looking at these individual profiles. For a more quantitative confirmation, we then shift each profile by its mean temperature $T_{m}$, and the six shifted profiles are plotted in the last panel on the right in Figure 3. These six shifted profiles overlap with each other perfectly and we only see one single curve there. These identical shifted profiles precisely confirm that the temperature values at same relative positions in individual periodic units are only different in respective mean temperatures and the variation fashion is identical. Furthermore, the mean temperature values calculated along these six profiles are listed in Table 1. According to Equation (7), these mean temperatures can be related to the mean value at x/D=0.6 by

(11) $$T_{m}\left(x_{i}\right)=T_{m}\left(x=0.6D\right)+\left(x_{i}-0.6D\right)T_{g}^{\prime },$$

with $x_{i}$ = 0.6D, 2.6D, 6.6D, 10.6D, 14.6D and 18.6D for the six profiles in Figure 3 (from left to right). The predicted values from this equation are also provided in Table 1 for comparison, and the excellent agreement convincingly confirms the validity of the temperature relation Equation (7) for such porous thermal flows.

## 4. Simulation Results and Discussion

The enhanced heat transfer performance in porous flow systems, in principle, benefits from the large solid–fluid contact area and the long, twisted flow path as the fluid passes through the microscopic porous structure. At the macroscopic, practical level, the apparent thermal conductivity is often used to quantify the overall thermal behaviors. The volume averaging analysis [21,51,52,53] can be applied to obtain the effective conductive tensor from the microscopic flow and thermal fields. Kuwahara et al. [21] further decomposed this conductivity tensor K into three parts

(12) $$K=K_{e}I+K_{tor}+K_{dis},$$

where $K_{e}$ is the stagnant conductivity, which is simply the volume average of the fluid and solid conductivities based on the porosity ϵ:

(13) $$K_{e}=\epsilon K_{f}+\left(1-\epsilon \right)K_{s}.$$

Please note that the expression for $K_{e}$ in Equation (13) is simply the coefficient for the isotropic part of the conductivity matrix K from the volume average analysis [21,51,52,53]; and that there are no assumptions involved on the microscopic porous structures.$K_{tor}$ and $K_{dis}$ are called the tortuosity and dispersion conductivity tensors, respectively. When the mean flow direction is along one of the coordinate directions, only diagonal elements are nonzero; and obviously the primary concerns are the diagonal elements in the global thermal gradient direction [21,53,54]. For the setup in Figure 1, we simply use $K_{tor}$ and $K_{dis}$ to denote the xx components of tensors $K_{tor}$ and $K_{dis}$; and they can be calculated from the simulated flow and temperature distributions in one periodic unit by [21,53]

(14) $$K_{tor}=-\frac{K_{s}-K_{f}}{hlT_{g}^{\prime }}\int_{\Gamma }n_{x}T_{f}d\Gamma ,$$

(15) $$K_{dis}=-\frac{\rho c_{pf}}{hlT_{g}^{\prime }}\int_{\Omega_{0}}\left(T-\bar{T}\right){\left(u-{\bar{u}}_{f}\right)}_{x}d\Omega ;$$

where $c_{pf}$ is the fluid specific heat, and the volume average temperature $\bar{T}$ and the intrinsic average velocity ${\bar{u}}_{f}$ are given by:

(16) $$\bar{T}=\frac{1}{lh}\left(\int_{\Omega_{0}}T_{f}d\Omega +\int_{\Omega_{1}}T_{s}d\Omega \right),\;{\bar{u}}_{f}=\frac{1}{\epsilon lh}\int_{\Omega_{0}}ud\Omega .$$

The subscript ${}_{x}$ denotes the *x* component of the corresponding vector.

In this section, we apply the boundary conditions in Section 2 to the periodic unit shown in Figure 1b, and examine the effects on the effective macroscopic conductivity coefficients $K_{tor}$ and $K_{dis}$ from several microscopic parameters, including the aspect ratio β=h/l, the Reynolds number $Re=\rho \sqrt{hl}U_{0}/\mu$, the interfacial thermal conductance *C*, and the porosity $\epsilon =1-\pi D^{2}/4hl$. We keep the unit area lh=90,000 constant in our next simulations, so that the Reynolds number Re is directly proportional to the mean flow velocity $U_{0}$, and the porosity ϵ depends purely on the solid diameter *D* and not affected by the aspect ratio β. The mean temperature at x=0 is set as $T_{0}=-0.05$ and the global thermal gradient is $T_{g}^{\prime }=0.1/l$. Please note that, due to the linearity and homogeneity of the energy equation Equation (3) and the associated thermal boundary conditions, the particular values of $T_{0}$ and $T_{g}^{\prime }$ used in the simulations do not affect the calculated conductivities $K_{tor}$ and $K_{dis}$ from Equations (14) and (15). To make this point clear, we consider a periodic unit under two situations: Situation (a) with mean inlet temperature $T_{0,a}$ and thermal gradient $T_{g,a}^{\prime }$; and Situation (b) with mean inlet temperature $T_{0,b}$ and thermal gradient $T_{g,b}^{\prime }$. If $T_{a}\left(x,y\right)$ is the solution in the periodic unit with Situation (a), the temperature field for Situation (b) should be $T_{b}\left(x,y\right)=T_{0,b}+\frac{T_{g,b}^{\prime }}{T_{g,a}^{\prime }}\left[T_{a}\left(x,y\right)-T_{0,a}\right]$. Clearly, $T_{b}\left(x,y\right)$ satisfies the energy equation Equation (3) and all temperature conditions in Equations (5), (8), (9) and (10). Substituting this expression of $T_{b}\left(x,y\right)$ in Equations (14) and (15) and considering $\int_{\Gamma }u_{x}d\Gamma =0$ for periodic structures, one can find that the $K_{tor}$ and $K_{dis}$ values remain the same for Situations (a) and (b). Other simulation parameters are kept the same as given in Section 3, unless otherwise mentioned. As for the initial state, we start simulations with a linear transition from $T_{0}$ at the inlet to $T_{0}+T_{g}^{\prime }l$ at the outlet for the temperature, and zero velocity for the flow. The results are taken when steady or quasi-steady states are established. The computer code for this work has been developed based on our programs used in previous publications [28,38,44,49,55,56], and all important elements involved in our simulations, including the LBM algorithms for flow and heat transfer, the no-slip boundary and conjugate interface treatments, as well as the mesh resolution selection, have been validated and confirmed in these previous studies.

### 4.1. Effects of the Aspect Ratio β and Reynolds Number Re

**Figure 4:**
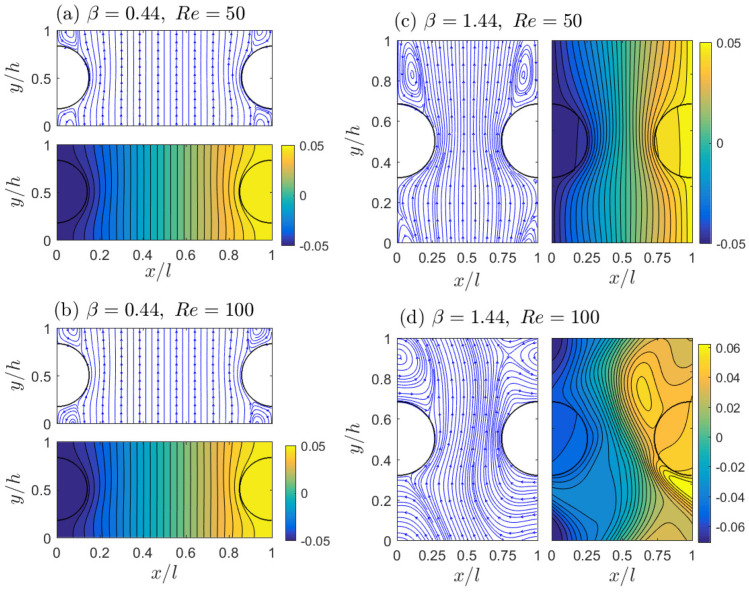
Simulation results of the flow streamlines and temperature distributions for β=0.44 (**a**,**b**) and β=1.44 (**c**,**d**) at Re = 50 (**a**,**c**) and 100 (**b**,**d**).

We start with simulations to study the effect of the aspect ratio β on the macroscopic thermal conductivity, which has not been well addressed in previous investigations. Here we set the porosity ϵ=0.85, and accordingly the solid domain diameter is D=131.11. Following previous studies [29,57,58], two representative Reynolds number values, Re=50 and 100, are considered in this section. Higher Reynolds numbers are possible for gas flows through porous media [59,60]. Our simulations cover a range of 0.25∼4 for the aspect ratio β; further increasing (>4) or decreasing (<0.25) of the β value will make the gap between the solid surfaces too small for accuracy and stable computations. The flows are always steady for all tested β values at Re=50 and for β≤1 at Re=100; however, the flow becomes unsteady for β>1 and Re=100. This transition is reasonable, since for a larger β, the gap between the solid surfaces becomes narrower, and the fluid velocity through this gap increases accordingly. This situation is similar to a flow jet coming out from a small opening shooting into a relatively large space. Compared to the condition with the same mean flow velocity (same Reynolds number) but a smaller aspect ratio β, the reduction in flow passage width is less significant (a wider gap between solid surfaces) and the fluid space after the gap is relatively limited. Figure 4 shows the streamlines and temperature distributions for two representative aspect ratios, β=0.44 and 1.44 at Re=50 and 100. For steady flows in Figure 4a–c, we see smooth and symmetric streamlines and isotherm contours. On the other hand, when the flow become unsteady in Figure 4d, these lines are distorted and less organized. Both for the steady and unsteady cases, thermal features discussed in Section 3 are noticed as well, including the relatively uniform temperature distributions inside the solid domains (due to the high solid conductivity $K_{s}$) and the smooth temperature transition across the solid–fluid interface (due to the large interface conductance *C*). Moreover, we see significant temperature variations along the unit boundaries for the unsteady system in Figure 4d. The variation fashions are similar on opposite edges. More specific, the temperature variations along the top and bottom boundaries appear identical, whereas the temperature is low on the left and high on the right. All these observations are consistent with the thermal boundary conditions in Equations (8) and (9).

**Figure 5:**
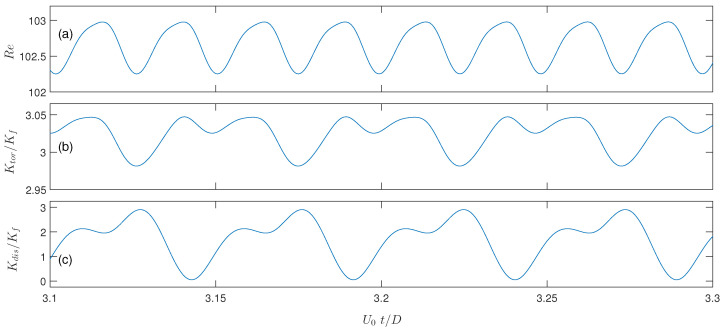
Variations of Re (**a**), $K_{tor}$ (**b**) and $K_{dis}$ (**c**) with time for the unsteady system with β=1.44 and Re≈100. The simulation time is normalized based on the mean velocity $U_{0}$ and solid cylinder diameter *D*.

For unsteady flow situations, the mean flow velocity $U_{0}$ and Reynolds number Re both vary with time, and it is difficult to achieve the time-averaged Reynolds number exactly at 100. For such situations, we accept the simulation results when the Re variation is limited in the range of 95∼105. The unsteady flow certainly affects the thermal field, and accordingly, the tortuosity and dispersion conductivities $K_{tor}$ and $K_{dis}$ calculated from Equations (14) and (15) also vary with time. Figure 5 displays the temporal oscillations of Re, $K_{tor}$ and $K_{dis}$ for Re=100 and β=1.44. The oscillation periods for $K_{tor}$ and $K_{dis}$ appear to be identical, as twice that for Re. In our next analysis, we use the average values over a variation period for the tortuosity and dispersion conductivities $K_{tor}$ and $K_{dis}$.

**Figure 6:**
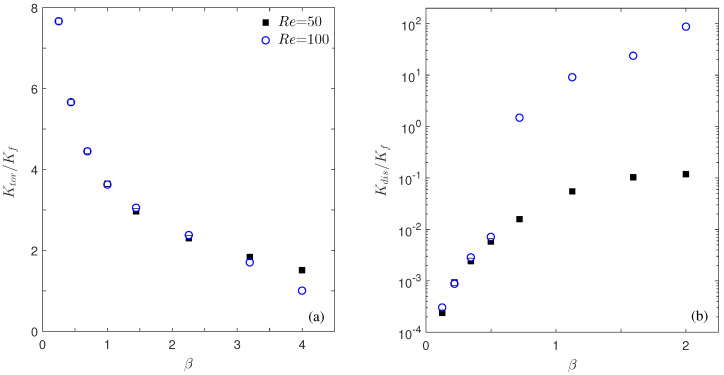
Plots of $K_{tor}$ (**a**) and $K_{dis}$ (**b**) changing with the aspect ratio β at two Reynolds numbers Re = 50 (black squares) and 100 (blue circles).

Figure 6 plots the apparent conductivities $K_{tor}$ and $K_{dis}$ changing with the aspect ratio β and Reynolds number Re. Clearly, even under the same porosity ϵ=0.85, the macroscopic conductivities can be greatly affected by the aspect ratio β, and the influence can be enhanced with a higher Reynolds number Re. The tortuosity conductivity $K_{tor}$ decreases with β in Figure 6a, which can be explained by looking at the $K_{tor}$ definition Equation (14) and the temperature distributions in Figure 4. Equation (14) can be interpreted as a weighted sum of the fluid temperature over the solid–fluid interface Γ, with $n_{x}$, the *x*-component of the normal vector n, as the weighting factor. For the system considered here, the left surface has $n_{x}>0$ and the right surface has $n_{x}<0$. On each semicircular surface, the portion around the horizontal centerline (the surface is approximately aligned in the vertical direction and thus it has a larger $|n_{x}|$ value) plays a more determinant role than the portions near the unit edges (the surface is approximately aligned in the horizontal and thus $|n_{x}|\approx 0$). Therefore, by comparing the temperatures on the parts around the horizontal centerline of the two semicircular surfaces in Figure 4, we can have a qualitative understanding of the $K_{tor}$ dependence on β: For a large β, the two semicircular surfaces are closer and the temperature difference on the closest portions becomes smaller, and this smaller temperature difference results in a smaller tortuosity conductivity, as observed in Figure 6a. The Reynolds number Re appears to be less influential on $K_{tor}$, except for the highly unsteady case with β=4. The weak influence of Re on $K_{tor}$ is consistent with that observed in Ref. [21].

Unlike the tortuosity conductivity $K_{tor}$, the dispersion conductivity $K_{dis}$ increases by orders with β in Figure 6b. Similarly, we attempt to interpret this observation by examining the $K_{dis}$ definition Equation (15) and flow and thermal fields in Figure 4. Clearly, Equation (15) represents the disorder level of the flow and thermal distributions in the periodic unit, since the integrand is simply the product of the temperature fluctuation $T-\bar{T}$ and the flow fluctuation ${\left(u-{\bar{u}}_{f}\right)}_{x}$. Therefore, the more disordered the flow and temperature distributions in the periodic unit, the larger $K_{dis}$ value will be obtained. This analysis is consistent with our general understanding of the thermal dispersion process and it explains the increasing trend of $K_{dis}$ with β and Re in Figure 6b as well. The similarity in flow and thermal fields between Re=50 and 100 for β≤1 steady systems suggests that $K_{dis}$ does not change much with Re there; however, for β>1, the Re=100 systems become unsteady and the flow and temperature distributions become significantly disordered, resulting in an abrupt increase in $K_{dis}$. We also notice that only the *x*-component of the flow fluctuation is involved in Equation (15). For the steady flows at β≤1, the nonzero *x* fluid velocity is limited in the small circulation areas near the four unit corners, whereas for large β values, the circulation region is large. The high Reynolds number Re=100 is also helpful in increasing the circulation size, and it can even make the *x* velocity component nonzero over almost the entire fluid domain for the unsteady flows with β>1. This is the reason that we see the $K_{dis}$ increase with β and Re by orders for β>1 in Figure 6b.

### 4.2. Effect of the Interfacial Conductance *C*

**Figure 7:**
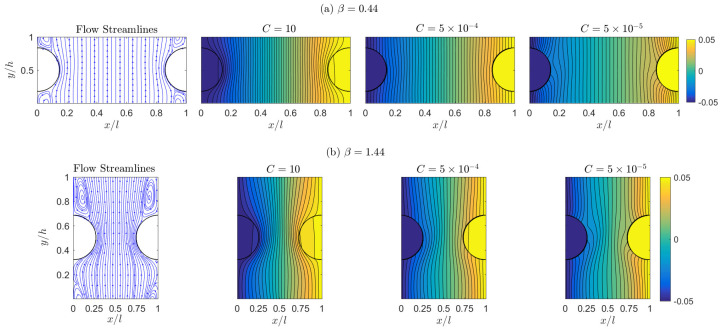
Simulation results of the flow streamlines (first column) and temperature distributions for different interfacial conductances C=10 (second column), $5\times 10^{-4}$ (third column), and $5\times 10^{-5}$ (last column) with the aspect ratios β=0.44 ((**a**) in the top row) and 1.44 ((**b**) in the bottom row) at Re = 50.

**Figure 8:**
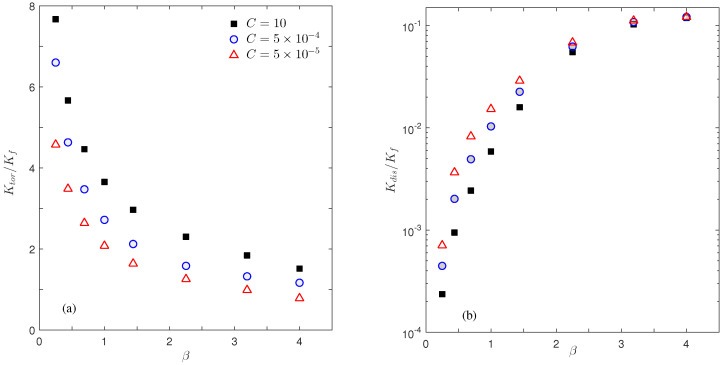
Plots of $K_{tor}$ (**a**) and $K_{dis}$ (**b**) changing with the aspect ratio β at three interfacial conductance values: *C* = 10 (black squares), $5\times 10^{-4}$ (blue circles), and $5\times 10^{-5}$ (red triangles). The Reynolds number is Re=50.

Next we shift our attention to the influence of the interfacial thermal conductance *C* on the apparent conductivities. Again this is an important topic [32,33], however, it has not been investigated adequately. In this part, we fix the porosity ϵ=0.85 and the Reynolds number Re=50 for simplicity, and test three interfacial conductance values C=10 (virtually no thermal resistance at the solid–fluid interface as observed in Figure 2b and Figure 4), $5\times 10^{-4}$, and $5\times 10^{-5}$. Figure 7 displays the flow and thermal distributions at these three conductance values with two aspect ratios β=0.44 and 1.44; and Figure 8 collects the calculated $K_{tor}$ and $K_{dis}$ conductivities in our simulations. The analysis in the previous section on the relationships between macroscopic conductivities and microscopic flow and thermal situations can still be applied here. Please note the change in interfacial conductance *C* does not affect the flow field and thus we can focus on the temperature response to different *C* values in Figure 7. As conductance *C* decreases, the solid domains become more insulated, resulting in a nearly constant temperature in each solid patch (i.e., the same colors and no isotherm contours in solid domains). With the solid part being insulated and thus its high-conductivity influence reduced, the fluid temperature around the solid domains exhibits a faster change along the thermal gradient direction. For example, the fluid temperatures near the solid surfaces at the narrowest gap location in Figure 7a are −0.0471 (left) vs. 0.0473 (right) for C=10, −0.0364 vs. 0.0366 for $C=5\times 10^{-4}$, and −0.0251 vs. 0.0254 for $C=5\times 10^{-5}$. As discussed in the previous section, the large temperature difference at C=10 introduces a larger tortuosity conductivity $K_{tor}$, and vise versa, as shown in Figure 8a. On the other hand, the dispersion conductivity $K_{dis}$ increases as we reduce the interfacial conductance *C*, and the *C* influence becomes negligible for large aspect ratios. This change should be attributed to the temperature change in circulation areas; however, due to the complexity in flow structures and temperature changes with *C* in Figure 7, we are not able to provide a direct qualitative explanation here. Overall, the influence of the interface conductance *C* on the apparent conductivities is less dramatic in Figure 8 than that from the unit aspect ratio β in Figure 6, but it cannot be neglected for accurate analysis.

### 4.3. Effect of the Porosity ϵ

**Figure 9:**
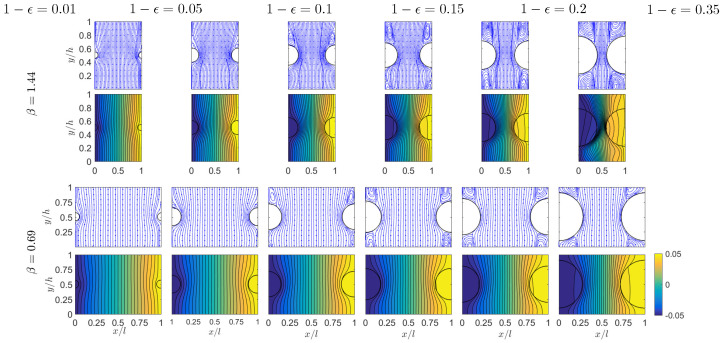
Simulation results of the flow streamlines (first and third rows) and temperature distributions (second and last rows) for different porosity values (labeled on top of each column) with interfacial conductance C=10 and Reynolds number Re = 50.

Unlike the effects of aspect ratio β and the interfacial conductance *C*, extensive studies have been conducted for the influence of the porosity ϵ on the macroscopic thermal behaviors of porous materials [21,61,62]. As mentioned in Section 1, previous pore-scale simulations usually set isothermal conditions on a pair of unit boundaries to create the thermal gradient and applied adiabatic conditions on other unit side surfaces [24,27]. Such artificial, unphysical treatments interfere with the natural heat transfer processes among microscopic periodic units, and the results from such boundary methods could be inaccurate or misleading [28]. In this section, we use the generalized periodic condition Equation (7) to examine the porosity effect on macroscopic conductivities $K_{tor}$ and $K_{dis}$. Our next simulations cover a range of porosity of ϵ=0.5∼0.99 for aspect ratios β = 0.69 and 1, and ϵ=0.65∼0.99 for and β=1.44. The Reynolds number is set at Re=50 and the interfacial conductance is fixed at C=10. Further reducing ϵ will increase the solid cylinder diameter *D* and thus make the gap between the solid surfaces very narrow (Figure 9). As discussed in Section 4.1, such a narrow gap may turn the flow unsteady for the large aspect ratio β=1.44, and also a finer spacial resolution is required to accurately capture the flow and thermal variations in the gap regions.

**Figure 10:**
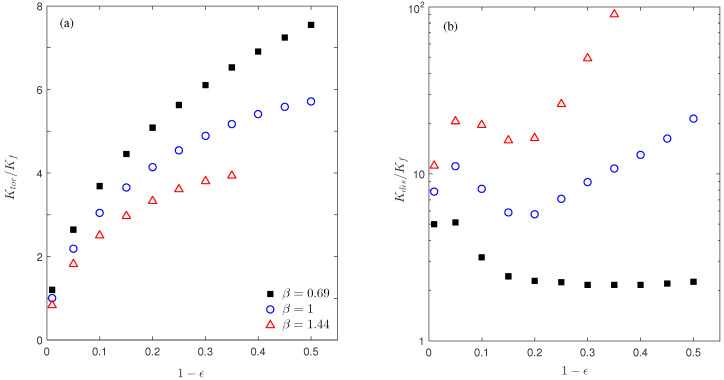
Plots of $K_{tor}$ (**a**) and $K_{dis}$ (**b**) changing with the solid fraction 1−ϵ at three aspect ratio values: β = 0.69 (black squares), 1 (blue circles), and 1.44 (red triangles). The Reynolds number is Re=50.

Results from this set of simulations are collected in Figure 9 for the flow and temperature distributions and Figure 10 for the calculated conductivities $K_{tor}$ and $K_{dis}$ at different porosity values. Please note that these figures are plotted in terms of the solid fraction 1−ϵ as in Ref. [21]. With the solid diameter *D* increase (from left to right in Figure 9), a pair of circulation vortices are developed in the wake region above the solid cylinder, and the vortex size grows gradually till it completely fills the vertical space between two cylinders. Meanwhile, the original relatively organized temperature field is gradually distorted by the increasing size of the solid domain. Such changes in the flow and thermal patterns therefore affect the macroscopic thermal behaviors as characterized by the tortuosity ($K_{tor}$) and dispersion ($K_{dis}$) conductivities (Figure 10). The continuous increase in $K_{tor}$ with the solid fraction 1−ϵ is mainly because of the larger area (length in our 2D system) of the solid–fluid interface Γ (see Equation (14)). This trend is similar to that observed in Ref. [21]; however, the $K_{tor}$ magnitude is smaller here. This can be attributed to the different solid domain shapes: For the same porosity, compared to the circular shape in this work, the square solid domain shape in Ref. [21] yields a longer interface length, and more profoundly, half of the interface has the local normal direction aligned the *x* direction. All these are favorable for a larger $K_{tor}$ according to its definition in Equation (14). As for the dispersion conductivity $K_{dis}$ in Figure 10b, in general, $K_{dis}$ increases with the solid fraction 1−ϵ; however, unlike the monotonic growth in Ref. [21], local maximum and minimum states are observed here. Due to the complexity in flow and temperature fields as well as their involvement in the $K_{dis}$ calculation, it is difficult to provide detailed insights and mechanism for the $K_{dis}$ variations. Nevertheless, this could be an interesting topic to explore in the future. The general increasing trend can be qualitatively related to the increasing size of the circulation region, which is the major contributor to $K_{dis}$ via the large *x* velocity component in this region. A similar analysis can be applied for the gentle variation and slow recovery of $K_{dis}$ for 1−ϵ=0.2∼0.5 at β=0.69 in Figure 10b, since the flow pattern remains almost unchanged in these systems (see the subplots for 1−ϵ=0.2 and 0.35 at β=0.69 in Figure 9). Finally, for the three curves with different aspect ratios, we see $K_{tor}$ is smaller but $K_{dis}$ is larger for a higher aspect ratio β. This agrees well with our findings and discussions for the aspect ratio effect on conductivity in Section 4.1 (see Figure 6).

## 5. Summary

In this study, we have first justified and validated the generalized periodic condition [21,28] for pore-scale simulations of thermal flows in porous media. Extensive simulations have then been carried out using a 2D porous model to investigate the influence of several microscopic parameters on macroscopic thermal conductivity. Among them, the effects of the aspect ratio of the periodic unit and the thermal conductance at the pore surface have not been addressed adequately in previous studies. Our results show that these microscopic properties can dramatically change the flow and thermal fields in the microscopic porous structure, and affect the apparent thermal performances of the porous materials at the macroscopic level. Therefore, these microscopic factors need to be considered carefully for more accurate and reliable simulation results, which are crucial for both fundamental research and practical applications. In addition, thorough discussions are attempted to qualitatively explore the relationship between the apparent conductivity at the macroscopic level and the complex thermal flow situations in the microscopic porous structure, and our analysis and findings could be helpful for a better understanding of the underlying thermal processes.

We are aware that several serious limitations exist for this study and one should not over interpret the results obtained from a specific system. The simple 2D porous model and the relatively low Reynolds numbers considered here may appear less realistic for practical systems; however, they are helpful for understanding the micro–macro relations and fundamental mechanisms involved in the complex thermal flow processes in porous materials. We have fixed the solid and fluid substance properties (conductivity, diffusivity, and the Prandtl number), which can certainly affect the apparent thermal behaviors as well. The 2D unit geometry adopted in this work is also symmetric along both the flow and thermal gradient directions; and the anisotropic effects could be an interesting topic for future research. The periodic conditions Equations (8) and (9) utilized in these calculations require the flow and thermal fields to be fully developed and thus they are not applicable to the entrance and development regions [63,64]. Moreover, in our simulations, we have assumed that the material properties are constant in the flow and heat transfer processes and thus steady or quasi-steady states can be established. In some situations, the microscopic porous structure may undergo a dynamic change due to swelling and erosion [65]; and caution must be taken for applying results from this study to such systems. Nevertheless, the boundary method, simulation results, and analysis discussions can be useful for the research and applications of porous thermal flows. The generalized periodic boundary condition, although presented in 2D, can be readily applied to three-dimensional pore-scale thermal flow simulations.

## Figures and Tables

**Table 1:** Comparison of the mean temperature values at different horizontal positions obtained from the simulation and predicted by Equation (11).

x/D	Calculated from Simulation Results	Predicted from Equation (11)
0.6	0.03847	-
2.6	0.13857	0.13847
6.6	0.33872	0.33847
10.6	0.53881	0.53847
14.6	0.73878	0.73847
18.6	0.93860	0.93847

## References

[1] Ingham D.B., Pop I. (2005). Transport Phenomena in Porous Media III.

[2] Ottenhall A., Illergard J., Ek M. (2017). Water Purification Using Functionalized Cellulosic Fibers with Nonleaching Bacteria Adsorbing Properties. Environ. Sci. Technol..

[3] Boules D., Sharqawy M.H., Ahmed W.H. (2021). Enhancement of heat transfer from a horizontal cylinder wrapped with whole and segmented layers of metal foam. Int. J. Heat Mass Transf..

[4] Bianco N., Busiello S., Iasiello M., Mauro G.M. (2021). Finned heat sinks with phase change materials and metal foams: Pareto optimization to address cost and operation time. Appl. Therm. Eng..

[5] Li Y., Gong L., Ding B., Xu M., Joshi Y. (2021). Thermal management of power electronics with liquid cooled metal foam heat sink. Int. J. Therm. Sci..

[6] Iasiello M., Mameli M., Filippeschi S., Bianco S. (2020). Simulations of paraffine melting inside metal foams at different gravity levels with preliminary experimental validation. J. Phys. Conf. Ser..

[7] Fu X., Viskanta R., Gore J. (1998). Measurement and correlation of volumetric heat transfer coeffcients of cellular ceramics. Exp. Therm. Fluid Sci..

[8] Wang G., Zhang Z., Wang R., Zhu Z. (2020). A Review on Heat Transfer of Nanofluids by Applied Electric Field or Magnetic Field. Nanomaterials.

[9] Chuan L., Wang X.D., Wang T.H., Yan W.M. (2015). Fluid flow and heat transfer in microchannel heat sink based on porous fin design concept. Int. Commun. Heat Mass Transf..

[10] Behrang A., Taheri S., Kantzas A. (2016). A hybrid approach on predicting the effective thermal conductivity of porous and nanoporous media. Int. J. Heat Mass Transf..

[11] Gong L., Wang Y., Cheng X., Zhang R., Zhang H. (2014). A novel effective medium theory for modelling the thermal conductivity of porous materials. Int. J. Heat Mass Transf..

[12] Song Y.S., Youn J.R. (2006). Evaluation of effective thermal conductivity for carbon nanotube/polymer composites using control volume finite element method. Carbon.

[13] Ye C., Huang H., Fan J., Sum W. (2008). Numerical Study of Heat and Moisture Transfer in Textile Materials by a Finite Volume Method. Commun. Comput. Phys..

[14] Chiappini D., Festuccia A., Bella G. (2018). Coupled lattice Boltzmann finite volume method for conjugate heat transfer in porous media. Numer. Heat Transf. Part A Appl..

[15] Guo Z., Zhao T.S. (2005). A lattice Boltzmann model for convection heat transfer in porous media. Numer. Heat Transf. Part B Fundam..

[16] Liu Q., He Y.L. (2017). Lattice Boltzmann simulations of convection heat transfer in porous media. Phys. A Stat. Mech. Its Appl..

[17] Azadi P., Farnood R., Yan N. (2010). FEM-CDEM modeling of thermal conductivity of porous pigmented coatings. Comput. Mater. Sci..

[18] Duan Y., He S., Ganesan P., Gotts J. (2014). Analysis of the horizontal flow in the advanced gas-cooled reactor. Nucl. Eng. Des..

[19] Zhou Y., Yan C., Tang A.M., Duan C., Shengshi D. (2019). Mesoscopic prediction on the effective thermal conductivity of unsaturated clayey soils with double porosity system. Int. J. Heat Mass Transf..

[20] Huang P., Zhao Y., Niu Y., Ren X., Chang M., Sun Y. (2018). Mesoscopic Finite Element Method of the Effective Thermal Conductivity of Concrete with Arbitrary Gradation. Adv. Mater. Sci. Eng..

[21] Kuwahara F., Nakayama A., Koyama H. (1996). A Numerical Study of Thermal Dispersion in Porous Media. J. Heat Transf..

[22] Yang P., Wen Z., Dou R., Liu X. (2016). Effect of random structure on permeability and heat transfer characteristics for flow in 2D porous medium based on MRT lattice Boltzmann method. Phys. Lett. A.

[23] Jeong N., Choi D.H. (2011). Estimation of the thermal dispersion in a porous medium of complex structures using a lattice Boltzmann method. Int. J. Heat Mass Transf..

[24] Zhao C., Dai L., Tang G., Qu Z., Li Z. (2010). Numerical study of natural convection in porous media (metals) using Lattice Boltzmann Methods (LBM). Int. J. Heat Fluid Flow.

[25] Liu Z., Wu H. (2016). Pore-scale study on flow and heat transfer in 3D reconstructed porous media using micro-tomography images. Appl. Therm. Eng..

[26] Liu Q., He Y.L., Li Q., Tao W.Q. (2014). A multiple-relaxation-time lattice Boltzmann model for convection heat transfer in porous media. Int. J. Heat Mass Transf..

[27] Ren Q., Chan C.L. (2016). Natural convection with an array of solid obstacles in an enclosure by lattice Boltzmann method on a CUDA computation platform. Int. J. Heat Mass Transf..

[28] Jbeili M., Zhang J. (2021). The Generalized Periodic Boundary Conditions for Microscopic Simulations of Heat Transfer in Heterogeneous Materials. Int. J. Heat Mass Transf..

[29] Ozgumus T., Mobedi M. (2015). Effect of Pore to Throat Size Ratio on Interfacial Heat Transfer Coefficient of Porous Media. J. Heat Transf..

[30] Yang P., Wen Z., Dou R., Liu X. (2017). Heat transfer characteristics in random porous media based on the 3D lattice Boltzmann method. Int. J. Heat Mass Transf..

[31] Grucelski A., Pozorski J. (2015). Lattice Boltzmann simulations of heat transfer in flow past a cylinder and in simple porous media. Int. J. Heat Mass Transf..

[32] Smith D.S., Alzina A., Bourret J., Nait-Ali B. (2013). Thermal conductivity of porous materials. J. Mater. Res..

[33] Fang W.Z., Gou J., Zhang H., Kang Q., Tao W.Q. (2016). Numerical predictions of the effective thermal conductivity for needled C/C-SiC composite materials. Numer. Heat Transf. Part A Appl..

[34] Jiang P.X., Li M., Lu T.J., Yu L., Ren Z.P. (2004). Experimental research on convection heat transfer in sintered porous plate channels. Int. J. Heat Mass Transf..

[35] Wang S.L., Li X.Y., Wang X.D., Lu G. (2018). Flow and heat transfer characteristics in double-layered microchannel heat sinks with porous fins. Int. Commun. Heat Mass Transf..

[36] Lu X., Zhao Y. (2019). Effect of flow regime on convective heat transfer in porous copper manufactured by lost carbonate sintering. Int. J. Heat Fluid Flow.

[37] Cengel Y. (2019). Heat and Mass Transfer: Fundamentals and Applications.

[38] Le G., Oulaid O., Zhang J. (2015). Counter-extrapolation method for conjugate interfaces in computational heat and mass transfer. Phys. Rev. E.

[39] Jbeili M., Zhang J. (2020). The Temperature Decomposition Method for Periodic Thermal Flows with Conjugate Heat Transfer. Int. J. Heat Mass Transf..

[40] Guo K., L L., Xao G., AuYeung N., Mei R. (2015). Lattice Boltzmann method for conjugate heat and mass transfer with interfacial jump conditions. Int. J. Heat Mass Transf..

[41] Patankar S.V., Liu C.H., Sparrow E.M. (1977). Fully Developed Flow and Heat Transfer in Ducts Having Streamwise-Periodic Variations of Cross-Sectional Area. ASME J. Heat Transf..

[42] Stalio E., Piller M. (2007). Direct Numerical Simulation of Heat Transfer in Converging-Diverging Wavy Channels. J. Heat Transf..

[43] Harikrishnan S., Tiwari S. (2020). Unsteady Flow and Heat Transfer Characteristics of Primary and Secondary Corrugated Channels. J. Heat Transf..

[44] Wang Z., Shang H., Zhang J. (2017). Lattice Boltzmann simulations of heat transfer in fully developed periodic incompressible flows. Phys. Rev. E.

[45] Li P., Zhang J. (2018). Simulating Heat Transfer through Periodic Structures with Different Wall Temperatures: A Temperature Decomposition Method. ASME J. Heat Transf..

[46] He X., Chen S., Doolen G.D. (1998). A Novel Thermal Model for the Lattice Boltzmann Method in Incompressible Limit. J. Comput. Phys..

[47] Guo Z., Shu C. (2013). Lattice Boltzmann Method and Its Applications in Engineering.

[48] Zhang J. (2010). Lattice Boltzmann method for microfluidics: Models and applications. Microfluid. Nanofluid..

[49] Wang Z., Colin F., Le G., Zhang J. (2017). Counter-Extrapolation Method for Conjugate Heat and Mass Transfer with Interfacial Discontinuity. Int. J. Numer. Methods Heat Fluid Flow.

[50] Succi S. (2001). The Lattice Boltzmann Equation.

[51] Whitaker S. (1967). Diffusion and dispersion in porous media. AIChE J..

[52] Whitaker S. (1969). Advances in theory of fluid motion in porous media. Ind. Eng. Chem..

[53] d’Hueppe A. (2011). Heat Transfer Modeling at an Interface between a Porous Medium and a Free Region. Ph.D. Thesis.

[54] de Lemos M.J.S. (2012). Turbulence in Porous Media: Modeling and Applications.

[55] Yin X., Zhang J. (2012). An Improved Bounce-Back Scheme for Complex Boundary Conditions in Lattice Boltzmann Method. J. Comput. Phys..

[56] Chen Q., Zhang X., Zhang J. (2015). Effects of Reynolds and Prandtl Numbers on Heat Transfer Around a Circular Cylinder by the Simplified Thermal Lattice Boltzmann Model. Commun. Comput. Phys..

[57] Dyga R., Placzek M. (2010). Efficiency of heat transfer in heat exchangers with wire mesh packing. Int. J. Heat Mass Transf..

[58] Alshare A.A., Strykowski P.J., Simon T.W. (2010). Modeling of unsteady and steady fluid flow, heat transfer and dispersion in porous media using unit cell scale. Int. J. Heat Mass Transf..

[59] He J., Zhang J., Shang H. (2019). Two-Phase Dynamic Modelling and Simulation of Transport and Reaction in Catalytic Sulfur Dioxide Converters. Ind. Eng. Chem. Res..

[60] Bonnet J.P., Topin F., Tadrist L. (2008). Flow Laws in Metal Foams: Compressibility and Pore Size Effects. Transp. Porous Media.

[61] Sumirat I., Ando Y., Shimamura S. (2006). Theoretical consideration of the effect of porosity on thermal conductivity of porous materials. J. Porous Mater..

[62] Zhao C.Y. (2012). Review on thermal transport in high porosity cellular metal foams with open cells. Int. J. Heat Mass Transf..

[63] Iasiello M., Cunsolo S., Bianco N., Chiu W.K.S., Naso V. (2017). Developing thermal flow in open-cell foams. Int. J. Therm. Sci..

[64] Suleiman A.S., Dukhan N. (2014). Forced convection inside metal foam: Simulation over a long domain and analytical validation. Int. J. Therm. Sci..

[65] Matias A.F.V., Coelho R.C.V., Andrade J.S., Araujo N.A.M. (2021). Flow through time–evolving porous media: Swelling and erosion. J. Comput. Sci..

